# Effects of Noise Exposure and Mental Workload on Physiological Responses during Task Execution

**DOI:** 10.3390/ijerph191912434

**Published:** 2022-09-29

**Authors:** Yurong Fan, Jin Liang, Xiaodong Cao, Liping Pang, Jie Zhang

**Affiliations:** 1 School of Aeronautic Science and Engineering, Beihang University, Beijing 100191, China; 2 Marine Human Factors Engineering Lab, China Institute of Marine Technology & Economy, Beijing 100081, China; 3 College of Aeronautics and Astronautics, Taiyuan University of Technology, Taiyuan 030024, China

**Keywords:** noise, ECG, EEG, eye movement, mental workload, task performance

## Abstract

Twelve healthy male students were recruited to investigate the physiological response to different noise exposure and mental workload (MW) conditions, while performing multi-attribute task battery (MATB) tasks. The experiments were conducted under three noise exposure conditions, with different sound pressure levels and sharpness. After adaptation to each noise condition, the participants were required to perform the resting test and the MATB task tests with low, medium, and high MW. The electroencephalogram (EEG), electrocardiogram (ECG), and eye movement data were obtained, during the periods when participants were in the resting and task taking state. The results showed that subjects’ physiological responses at rest were unaffected by noise exposure conditions. However, during the execution of MATB tasks, the elevated sound pressure level and increased sharpness were significantly correlated with increased mean pupil diameter and heart rate variability (HRV). These responses suggested that the human body defends itself through physiological regulation when noise causes adverse effects. If the negative effects of noise were more severe, this could damage the body’s health and result in a significant drop in task performance. The elevated mental demands led to increased stress on the subjects, which was reflected in a considerable increase in theta relative power. Either high or low MW was related with reduced saccade amplitude and a decrease in weighted task performance, indicating an inverted U-shaped relationship between workload level and work performance.

## 1. Introduction

Acoustic noise, defined as disturbing, unpleasant, or unwanted sound, is now considered a pervasive pollutant that affects public health, well-being, and mortality worldwide [1,2]. A recent meta-analysis of studies from 1993 to 2019 found that the prevalence of occupational noise-induced hearing loss was 21.3% in China [3]. More than a quarter of current workers have been exposed to occupational noise in the United States [4]. In its 2020 environmental noise report [5], the European Environmental Agency (EEA) stated that, annually, chronic noise exposure causes 22 million people to suffer high annoyance, 6.5 million people to suffer high sleep disturbance, and 12,500 children to suffer learning impairment in school.

The negative impact of noise on human health is related to the fact that noise acts as an environment stressor and influences physiological, psychological, and behavioral processes [6,7]. Based on Babisch’s classical noise effect reaction scheme, both direct and indirect pathways for adverse health effects of noise have been theorized [8,9]. On one hand, the direct pathway is initiated as a result of the instantaneous interaction between the auditory nerve and structures of the central nervous system [10]. On the other hand, low-level noise exposure will activate the indirect pathway, resulting in the disturbance of activities, sleep and communication, and negative emotional responses [11]. Thus, there is sufficient scientific evidence that exposure to noise is associated with various negative health effects [12,13].

Noise is influenced by various properties of the environment and the sound source, which contribute to the different qualities of the perceived sound. The sound pressure level (SPL) has long been considered a critical factor in response to the effects of noise with its correlation to perceived sound loudness or sound intensity. A recent study showed that subjects’ physiological responses could change significantly when noise levels were above 70 dB (A) [14]. A previous study systematically summarized the possible health damage caused by exposure to different noise intensities [15]. A noise level that increases from 35 to 65 dB (A) can induce annoyance, which can alter the sleep–wake cycle, in addition to nonauditory effects. Noise ranging from 66 to 85 dB(A) can cause activation alarms, psychological and neurovegetative effects, and hearing damage. Noise between 85 and 115 dB (A) can significantly trigger acoustic and psychological disturbances, even in other target systems. A large case-control study found a 1.6% significantly higher risk of heart failure for every 10 dB (A) increment in aircraft noise [16]. A systematic review suggested that the risks of hypertension caused by noise levels of 80–85 dB (A) and 85–90 dB (A) were 1.77 and 3.50, respectively [17,18].

With the increasing cognitive demands of modern technology, mental workload (MW) has become an increasingly important topic in ergonomics [19,20]. One of the most exciting research topics is the relationship between MW and human performance [21,22,23]. Brookhuis and Waard [24] believed that overload or underload could both be considered suboptimal MWs. In other words, mental underload can adversely affect task performance just as much as mental overload, causing performance degradation, attentional lapses, and errors [20]. Therefore, an optimal range of MW contributes to the best performance and efficiency, for which the measurement of physiological parameters enables objective and real-time assessment of MW [25]. The change in MW requires cognitive resources to maintain human performance, affecting various physiological activities, such as cardiac activity, brain activity, eye movements, and metabolic activity [26,27].

An electrocardiogram (ECG) is used to measure the electrical activity of the heart and generate a series of repeating electrical signals. The heart rate (HR) and heart rate variability (HRV) can be used to analyze the effects of noise levels on the autonomic nervous system. An increase in HR was discovered in 88.9% of subjects exposed to a noise level of 90 dB intensity for 10 min [28]. Individual daytime noise exposure was found to be connected to immediate variations in HRV [29]. Furthermore, a multi-sensor study revealed that personal noise exposure was correlated with a concurrent increase in HRV [30]. Several systematic reviews concluded that HR and HRV were the most commonly used ECG measurements to gain insight into MW. HR increases with increasing task demands [31,32], task difficulty [33], and memory loads [34]. Gao et al. [35] discovered that HRV increased in a task with high complexity. Fallahi et al. [36] studied the effects of MW on physiological reactions during traffic density monitoring. Their findings suggested that operators’ HRV metrics were significantly impacted by rising traffic density.

An electroencephalogram (EEG) is the recording of the brain’s spontaneous electrical activity over a period of time, which can be decomposed into the following five frequency bands: delta (1–3 Hz), theta (4–7 Hz), alpha (8–13 Hz), beta (14–30 Hz), and gamma (>30 Hz) [37]. EEG has been considered one of the most commonly used physiological measures of MW in air traffic control studies [38]. A recent study used EEG to assess cognitive performance under construction noise conditions, which showed a negative correlation between cognitive function and noise exposure [39]. Beta and gamma powers in the left temporal and right prefrontal cortex were found to distinguish between the focused and distracted state of construction workers caused by noise, particularly in channels T7 and AF4 [40]. Alpha band activity has been shown to be sensitive to MW during multi-attribute tasks and air traffic control tasks [41,42]. Wilson [43] observed a decrease in alpha power during the takeoff and landing phases of flight. Beta power and theta power were both positively associated with MW [44,45].

Measurements based on different kinds of eye movements, such as blink rate, fixation duration, pupil diameter, and saccade-related measures, have been used to derive metrics of MW [46]. The blink rate and saccadic peak velocity were found to decrease with the increase in visual and cognitive demands during the air traffic control simulated tasks [33,47]. When performing nuclear power plant operation procedures [48] and dealing with artificial surrounding created by computers [49], the pupil diameter was significantly larger with a higher MW. A few researchers have studied the association between noise exposure and eye movements. In a reading comprehension task, sound pressure levels that elevated from 50 dB(A) to 70 dB(A) did not affect task performance or higher level gaze behavior, such as saccade amplitude, fixation duration, and regressions [50]. Liao et al. [51] found a linear relationship between sound loudness and pupillary dilation response, which reflected the subjective salience of sounds.

In summary, numerous physiological measurements can be used to study the effect of noise exposure and MW on physiological reactions [29,40]. It is increasingly recognized that the combination of multiple measures can provide better coverage of more comprehensive human responses than relying on only one single measure [52]. Although there has been considerable discussion of the association between sound pressure levels and physiological reactions, few researchers have focused on the effects of other noise quality parameters, such as sharpness. To address these issues, we coupled the multi-attribute task battery (MATB) task tests with the ECG, EEG, and eye movement data collected to study the physiological responses to different noise parameters and MW levels and determine their effects on task performance.

## 2. Materials and Methods

### 2.1. Study Design

The experiment was conducted in an enclosed environment chamber with the independent variables of noise exposure conditions and MW levels. Three different noise exposure conditions were designed, with varying SPLs and noise sharpness. Each subject was exposed to one noise condition per experiment, and the order of the three noise exposure conditions was balanced by the Latin square method. The MATB task [53] developed by the National Aeronautics and Space Administration (NASA) was carried out to evaluate the subjects’ multitasking cognitive performance with different MWs, which consisted of the following three subtasks: system monitoring, tracking monitoring, and resource management. During each noise exposure condition, the recruited participants were required to perform MATB task tests with three different mental demands. The number of triggers for each subtask in twelve mins was set to 2, 16, and 36 at low mental workload (LMW), medium mental workload (MMW), and high mental workload (HMW), respectively. The ECG, EEG, and eye movement data were synchronously recorded while subjects were at rest and engaged in the tasks. In addition, subjective MW was evaluated by the NASA Task Load Index (NASA-TLX) scale [54] during the task tests. The research protocol was approved by the Institutional Review Board (IRB) of Beihang University.

Twelve healthy male college students with a mean age of 23.25 ± 2.31 and a mean body mass index (BMI) of 22.57 ± 1.95 kg/m were recruited for this experiment. The statistical power was 0.8, as calculated by the G*power software (version 3.1.9.7), when the effect size was assumed to be 0.4. All participants signed informed consent forms and passed the psychological personality tests. None of the participants had any history of disorders or previous exposure to occupational noise. The results of the hearing screening tests showed that all subjects had normal hearing. After adequate training and practice, the participants were asked to have good sleep, maintain a good mood, and consume no drinks containing alcohol, caffeine, or tea during the experiment. All subjects were blinded to the experimental conditions and completed the experiment without dropping out.

### 2.2. Experimental Conditions

Generally, the logarithmic decibel scale is used to express the SPL [55]. The equivalent A-weighted SPL (L_Aeq_) in decibels is typically used to represent the energy-averaged sound intensity over a period of time [56,57]. Sound sharpness (acum) was considered an essential part of sound quality and was regarded as a psychoacoustic hearing sensation to be considered separately [58]. The sharpness of the sound signal is closely related to the proportion of high-frequency components, which can reflect the human subjective feeling of sound harshness. The sound sharpness can be reduced by mixing low-frequency components, which opens up possibilities for sound quality design [58,59]. The experimental study was carried out under the following three noise exposure conditions: the reference noise condition (N85-S1), the modified noise with lower SPL (N80-S1), and the modified noise with lower SPL but with worse sound quality (N75-S2). N85-S1 represents the actual noise signals collected from the ventilation equipment of a ship cabin, with a calculated SPL of 85 dB(A). In comparison to N85-S1, the SPL of N80-S1 was reduced to 80 dB(A), while the sharpness remained unchanged. N75-S2 was designed to have an SPL of 75 dB (A), but with increased noise sharpness.

**Figure 1:**
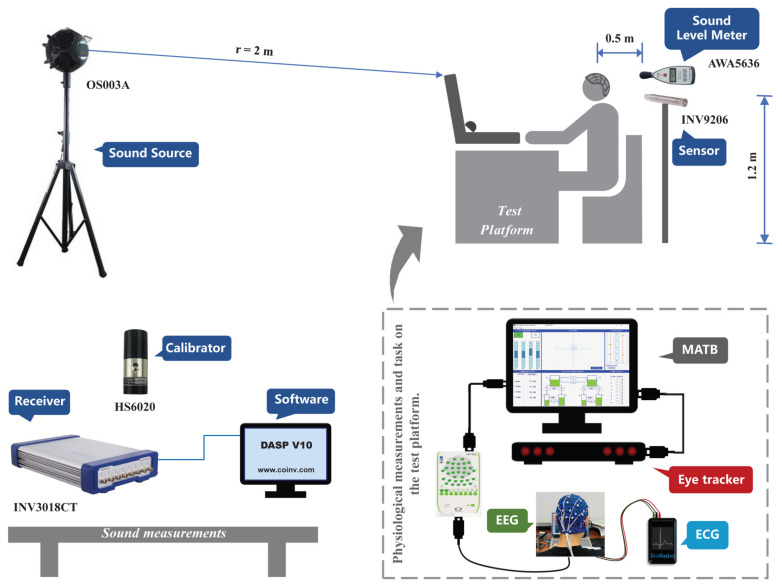
Schematic diagram of the experimental platform.

During the experiment, the designed noise files were played through a nondirectional sound source (OS003A, BSWA Technology Co., Beijing, China) placed in a fixed position in front of the subject, as shown in Figure 1. The INV9202 sound pressure sensor (COINV, Beijing, China) is a high-performance electret capacitive test sensor specially used for acoustic signal measurement. In the frequency range of 20 Hz–20 kHz, its technical indicators meet the first-class accuracy of IEC61672 [60] and GB/T3785. A sound level meter (AWA5636, Aihua Instruments Co., Hangzhou, China) was placed near the left ear of the subject to perform the real-time measurement of SPL. The sound level meter was calibrated once a day by an acoustic calibrator (HS6020) and has a measurement range of 40–130 dB(A), with a measurement accuracy of ±1.0 dB (A). A 24-bit high-precision USB receiver (INV3018CT, COINV) operated in conjunction with the dynamic test and analysis software (DASP-V10, COINV) for data acquisition and signal processing. An integrated indoor environmental quality tester (MI6401, METREL, Eckental, Germany) was used to measure indoor thermal parameters (Table 1).

The acoustic and thermal parameters were continuously measured during the experiment, as shown in Table 2. The measured noise parameters during the experiment were basically consistent with the designed level. The predicted mean vote (PMV) model can be used to predict the thermal sensation of the subject using a 7-point scale from −3 (cold) to +3 (hot). The metabolic rate of the human body was set at 90 W/m^2^ when performing the light manual test [61]. According to the ANSI/ASHRAE Standard 55–2010 [62], the estimated clothing insulation for a subject was 0.90 clo, with an ensemble of briefs, ankle-length athletic socks, shoes, thick straight trousers, a thick sleeveless vest, thick long-sleeve, and sitting in a metal chair. The estimated PMV ranged from −0.117 to 0.375, indicating that that the experiment was conducted in a thermally neutral environment.

### 2.3. Physiological Measurement

Table 3 provides an overview of the metrics for the physiological parameters that were measured. The ECG signals were recorded at a sampling rate of 250 Hz with the Bio-Radio wireless physiological monitor. Based on the QRS waves of the ECG signal extracted by the BioCapture physiological monitoring system, the RR interval of each heartbeat cycle can be obtained. HRV represents the fluctuation in the time intervals between adjacent heartbeats, which is valuable for exploring the noise effects on the autonomic nervous system. To determine the physiological reactions to noise exposure and MW, statistical analysis of HRV parameters, including SDNN, RMSSD, and pNN50, was performed.

The NeuroScan Grael system (NeuroScan, EI Paso, TX, USA) was used to capture thirty-two EEG electrodes based on the ten-twenty international system of electrode placement [63]. The vertical and horizontal electrooculogram (EOG) electrodes were positioned near the subject’s eyes. During the experiment, the EEG signals were sampled at a rate of 1024 Hz, and the online reference electrode (REF) was located at the midpoint of the CZ and CPZ electrodes. The GND electrode was set as the ground electrode, and the impedance of all channels was controlled so as to be below 5 kΩ. The open software EEGLAB v14.1.1b was utilized to perform data preprocessing and metric calculations of EEG signals. The averages of M1 and M2 were the reference electrodes during offline processing, which were located at the mastoid position behind the left and right ears, respectively. Then, the re-reference signals were filtered by the finite impulse response (FIR) bandpass filter, where the cutoff frequency of the high-pass filter was set to 1 Hz and the cutoff frequency of the low-pass filter was set to 30 Hz. Furthermore, the independent component analysis (ICA) method and the automatic EEG artifact detector ADJUST were used to rectify the EEG signals. The delta, theta, alpha, and beta frequency bands of the processed EEG data during one-second sampling intervals were used to extract the relative power, which was calculated as a ratio between the power of each band and the power of the entire band.

Eye movement data were recorded by an eye-tracking device (aSee A3, 7invensun) with tracking technology of pupil center corneal reflection (Pupil-CR) [64]. The sampling frequency of aSee A3 is 60 Hertz (Hz), which means that a sample is registered once every 16.667 ms [65]. The accuracy of aSee A3 is 0.7 degrees, which reflects the average error between the actual position of the line of sight and the position of the line of sight captured by the eye tracker. Before recording, the subject was 65 cm away from the eye tracking device and was asked to complete the nine-point calibration of the right and left eyes to ensure data quality. The eye movement metrics were then calculated by the analysis software (aSee Studio software, 7invensun), including fixation duration, saccade amplitude, saccade velocity, and mean pupil diameter.

### 2.4. Experimental Procedure

**Figure 2:**
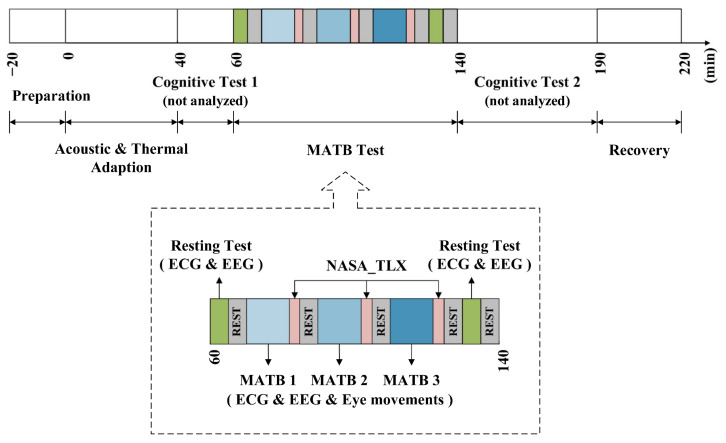
Flowchart of the experiment procedure.

**Figure 3:**
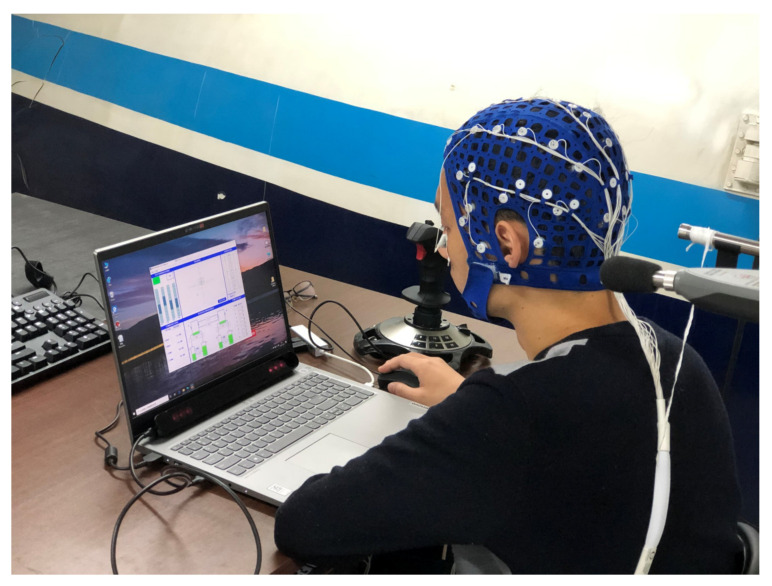
Experimental scene of the subject wearing the physiological monitoring equipment.

The experiments were conducted in November 2020 in morning (8:00–12:00) and afternoon (14:00–18:00) sessions from Monday to Saturday. Prior to the formal experiment, pretraining was conducted and each subject was familiar with how the MATB task was operated. As shown in Figure 2, the formal experimental procedure consisted of the following five phases: preparation (20 min), environmental adaptation (40 min), cognitive test 1 (25 min), MATB task test (75 min), and cognitive test 2 (60 min). During the preparation phase, each subject washed and blow-dried his hair for EEG measurement in a waiting room. After playing the specified noise file, the experiment proceeded to the noise adaptation phase, during which the experimenter wore the physiological monitoring equipment for the subject. Throughout the experiment, none of the subjects felt uncomfortable while wearing the physiological assessment devices. Then, each subject performed the resting test and the task test, during which EEG, ECG, and eye movement data (only during the task test) were collected simultaneously. The 5-min resting tests were performed both before and after the task test, which required the subject to alternately close and open their eyes for 30 s each. All subjects performed three 12-min MATB tasks with varying levels of MW in a sequence designed by the Latin square method during the task test. Each MATB task was followed by the completion of the NASA-TLX scale and then a 5-min break. Figure 3 shows the experimental scene where the required physiological parameters were recorded by the EEG, eye tracker and ECG. The focus of this study was the subjects’ physiological responses while performing the MATB tasks.

The generalized additive mixed-effects model (GAMM) analyses were carried out by the statistical software R (version 3.6.1, Vienna, Austria) to study the fixed effects of noise exposure and MW on physiological parameters, treating the subject as a random effect, as shown in Equations (1) and (2). The experimental conditions were treated as dummy variables. The physiological parameters collected from the ECG, EEG, and eye movements were used for the GAMM analysis. When the *p*-value was less than 0.05, the differences were considered statistically significant.

(1) $$y=\beta_{1}+\beta_{2}\left(Noise1\right)+\beta_{3}\left(Noise2\right)+\beta_{4}\left(MMW\right)+\beta_{5}\left(HMW\right)+b+e,$$

(2) $$y=\beta_{1}^{*}+\left(-\beta_{2}\left(Noise0\right)\right)+\beta_{3}^{*}\left(Noise2\right)+\left(-\beta_{4}\left(LMW\right)\right)+\beta_{5}^{*}\left(HMW\right)+b^{*}+e^{*},$$

where *y* stands for the physiological reaction index, which includes ECG, EEG, and eye movements; *β*_1_ and $\beta_{1}^{*}$ are fixed intercepts; *β*_2_ and *β*_3_ are the fixed effects of N80-S1 and N75-S2 relative to the reference N85-S1, respectively; $\beta_{3}^{*}$ is the fixed effect of N75-S2 relative to N80-S1; *β*_4_ and *β*_5_ are the fixed effects of MMW and HMW compared to LMW, respectively; $\beta_{5}^{*}$ is the fixed effect of HMW relative to MMW; *b* and $b^{*}$ are the random effects of participants; and the residuals are e and $e^{*}$.

## 3. Results

The GAMM results indicated that the individuals’ physiological parameters during rest were unaffected by noise exposure. When performing MATB tasks under different noise exposure conditions and MWs, the GAMM results for ECG, EEG, and eye movement metrics are shown in Table 4, Table 5 and Table 6, respectively.

### 3.1. Effects of Noise Exposure

**Figure 4:**
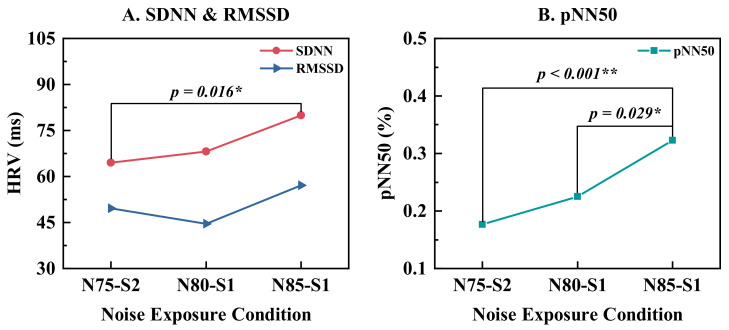
ECG metrics during task execution under three noise exposure conditions. * (*p* < 0.05), ** (*p* < 0.01).

As shown in Figure 4, both the SDNN and pNN50 increased significantly with enhanced noise intensity. This indicated that the HRV metrics were significantly higher when performing tasks with higher noise exposure, indicating that the body tended to be more self-protective through promoting the parasympathetic nervous system’s activity. The organism would use less resources and be more adaptable to greater noise levels because of the natural physiological defense. No statistically significant change in the relative powers of EEG was observed under varying noise exposure conditions (Table 5). The alpha relative power was modestly lower under N75-S2 than under N80-S1 (*p* = 0.066). This suggested that the subjects tended to be slightly stressed when sharpness increased.

**Figure 5:**
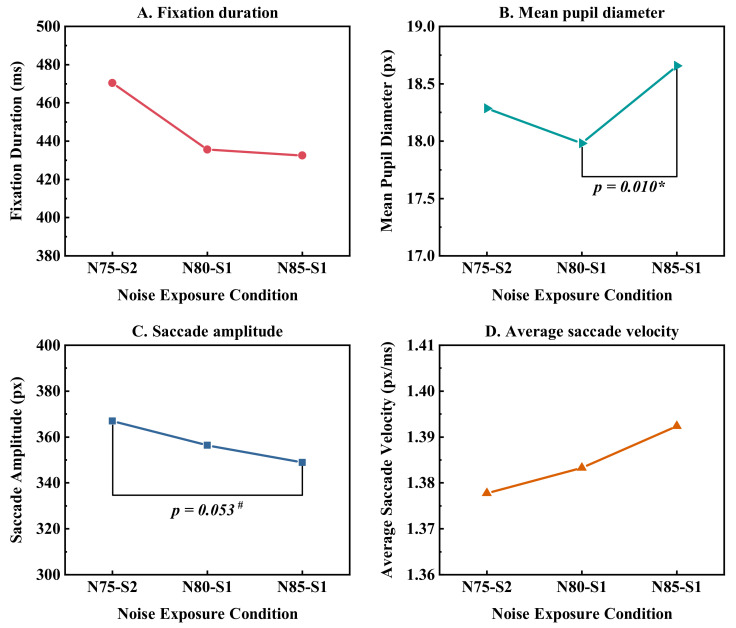
Eye movement metrics during task execution under three noise exposure conditions. # (*p* < 0.1), * (*p* < 0.05).

As illustrated in Figure 5, with the increase in SPL from 75 dB (A) to 85 dB (A), the fixation duration and saccade amplitude had a trend of gradual decline, the average saccade velocity had a trend of gradually increasing, and the mean pupil diameter took on a downward and then upward trend. The increase in the mean pupil diameter between N80-S1 and N85-S1 was statistically significant. The saccade amplitude decreased slightly from N75-S2 to N85-S1 (*p* = 0.053). As the SPL decreased from 85 dB (A) to 80 dB (A), the mean pupil diameter of the subjects decreased, suggesting that during the task, the body had less intraocular light intake and that visual information was more readily available. When exposed to SPL of 75 dB (A) but with increased sharpness, there was a small increase in mean pupil diameter, together with a slower saccade velocity. This implied that the elevated sharpness could result in the body struggling to acquire information and requiring more visual search effort.

### 3.2. Effects of Mental Workload

**Figure 6:**
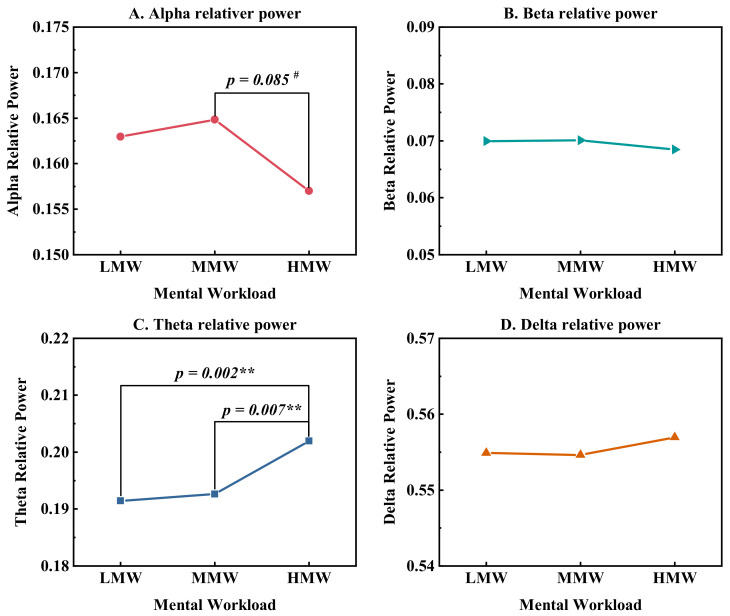
EEG relative band powers at three MW levels. # (*p* < 0.1), ** (*p* < 0.01).

There were no significant differences in HRV metrics between different MWs (Table 4). As shown in Figure 6, the subjects had significantly higher theta relative power at HMW than at lower MW levels, but no statistically significant difference was discovered in other frequency bands. The alpha relative power at HMW was slightly lower (*p* = 0.085) than that of MMW. This suggested that the subjects tended to be more depressed and stressed when performing HMW tasks in noisy environments.

**Figure 7:**
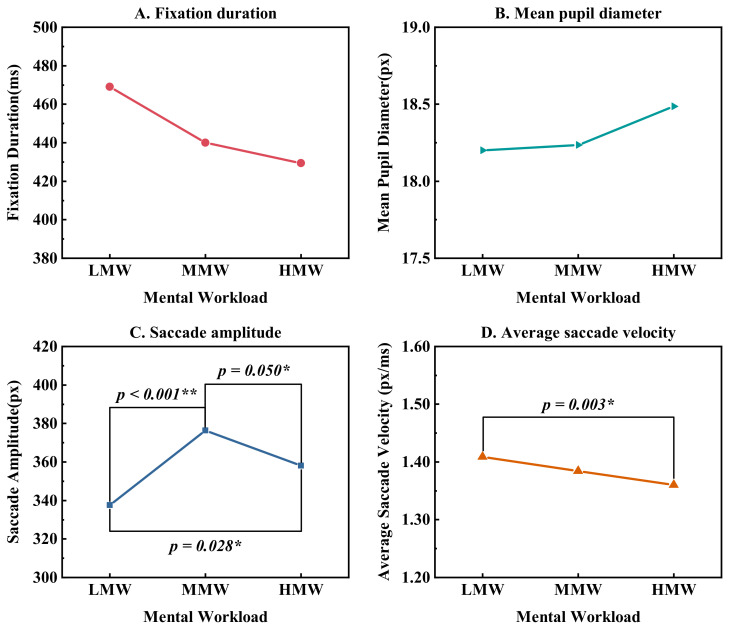
Eye movement metrics at three MW levels. * (*p* < 0.05), ** (*p* < 0.01).

**Figure 8:**
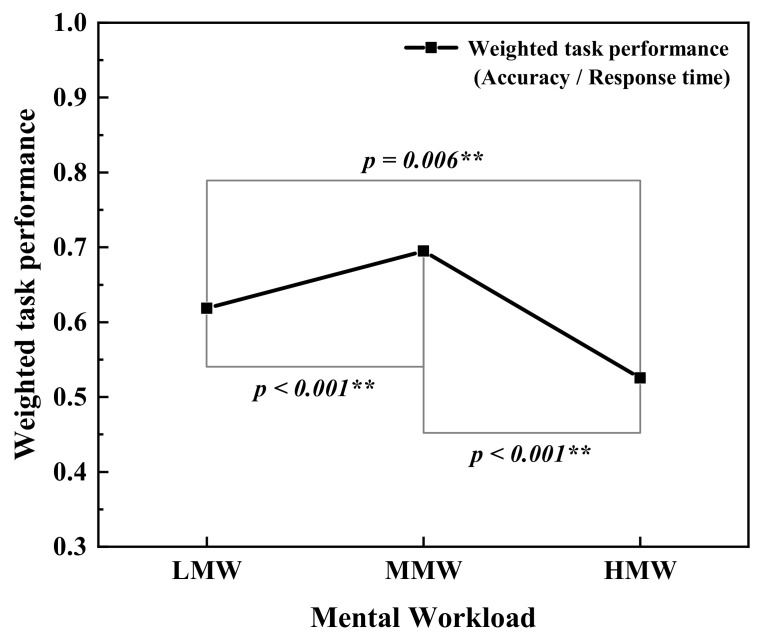
Weighted performance of MATB tasks at three MW levels. ** (*p* < 0.01).

As depicted in Figure 7, the average saccade velocity decreased significantly with the increased workload. The fixation duration also reduced slightly with the increased MW. The mean pupil diameter was slightly larger at HMW. This indicated that as the MW increased, the subjects needed to use more visual resources to complete tasks. However, the excessive task stimulus could diminish the visual search capability, making it difficult to capture information quickly and accurately, so that the ability to complete the MATB task could be impaired at HMW. This was manifested as an increase in the saccade amplitude from LMW to MMW and then a decrease from MMW to HMW, which helps explain the inverted U-shaped association between task performance and MW, as shown in Figure 8.

### 3.3. Correlation Analyses

**Figure 9:**
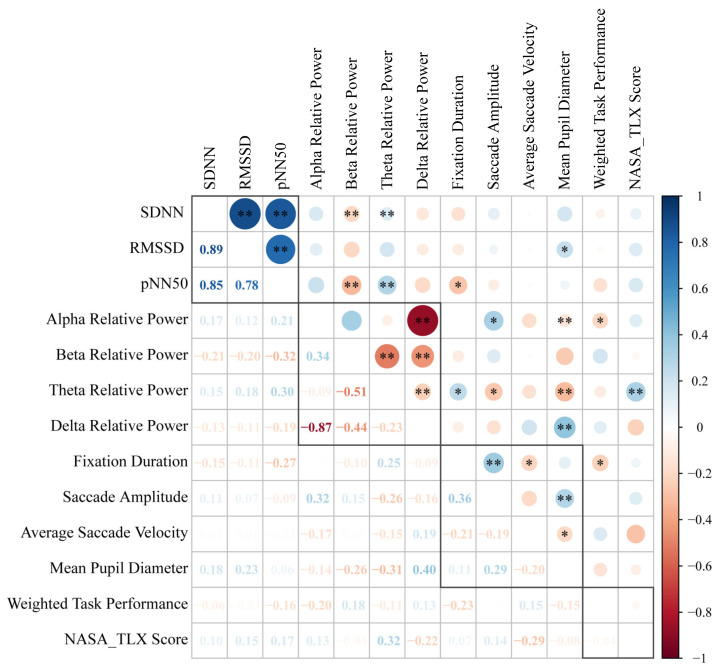
Correlation matrix heatmap between physiological parameters and task performance during the MATB tasks. * (*p* < 0.05), ** (*p* < 0.01).

Correlation analyses were further conducted using Pearson’s method [66] to determine the relationship between physiological responses and task performance during the MATB task tests. The analyzed performance metrics included the weighted task performance (average ratio of accuracy to response time) and the NASA-TLX score. Higher NASA-TLX scores indicate increased subjectively evaluated MW. Figure 9 displays the correlation matrix between different physiological parameters and task performance metrics. The circles (upper matrix elements) and numbers (lower matrix elements) with low correlation coefficients are lighter in color and smaller in size, while the circles and numbers with high correlation coefficients are darker in color and larger in size. The asterisks in the circles of the upper matrix indicate the significance level. As shown in Figure 9, there are strong positive correlations between SDNN and RMSSD (r = 0.89, *p* < 0.001), SDNN and pNN50 (r = 0.85, *p* < 0.001), and RMSSD and pNN50 (r = 0.78, *p* < 0.001). Therefore, the EEG metrics could be considered highly correlated. For EEG metrics, significant negative correlations were found between alpha relative power and delta relative power (r = −0.87, *p* < 0.001), beta relative power and theta relative power (r = −0.51, *p* = 0.001), and beta relative power and delta relative power (r = −0.44, *p* < 0.001). The duration of fixation has a weak positive correlation with the saccade amplitude (r = 0.36, *p* < 0.001). For correlations between physiological responses and MATB task performance, a weak positive correlation was observed between NASA-TLX scores and theta relative power (r = 0.32, *p* = 0.004), which suggests that heavier MW levels can lead to higher levels of depression and stress. Furthermore, the weighted task performance shows both a positive correlation with alpha relative power (r = 0.48, *p* < 0.001) and a negative correlation with delta relative power (r = −0.41, *p* < 0.001). These results suggest that better task performance is associated with less stress and fatigue. A possible explanation is that excessive stress could deplete the subject’s mental resources needed to fulfill the complicated tasks, thus leading to worse performance.

## 4. Discussion

### 4.1. Comparison with Previous Studies

The findings revealed that additional effort was taken to complete the MATB tasks under adverse conditions, including higher noise intensity, increased noise sharpness, and demanding workload. In addition, physiological responses were more significant during task execution than during rest, indicating that greater effort was made to cope with adverse conditions when performing the tasks.

In this study, elevated noise intensity was found to be significantly associated with increased HRV, which is consistent with many previous studies [29,30,67]. Lee et al. [68] found that the frequency-domain HRV parameters increased significantly with elevated noise intensity from 23 dB (A) to 80 dB (A). It is possible that increased sympathetic nervous system activity was accompanied by increased acute noise levels. In addition to noise intensity, we also found that the increase in HRV metrics could be alleviated by improved sound quality. The mean RMSSD even decreased at N80-S1 compared with N75-S2. The alpha relative power was modestly higher, and the mean pupil diameter was also found to be smaller under the N80-S1 condition, which implied reduced effort and lower emotional arousal when exposed to lower noise sharpness. This indicates that improved sound quality could weaken the fight-or-flight reaction, a physiological arousal that appears when the body perceives stress/dissatisfaction, resulting from adverse conditions.

The theta relative power was observed to increase significantly with the increased MW, which was consistent with the research findings for air traffic control tasks [34]. Similarly, Fairclough et al. [69] also concluded that the theta relative power increased significantly at higher task demands. The saccade amplitude showed a trend of first increasing and then decreasing with increasing task workload. The variations in saccade amplitude can be explained by the relationship between work demand and visual search effort. Fewer visual stimuli required narrow fixation areas and shorter visual search paths, indicating less visual search effort under the LMW. As visual stimuli increased, the subjects’ field of vision expanded, and the visual effort to extract key information was balanced with task demands. The subjects achieved the optimal visual search efficiency and task performance with moderate visual stimuli. As visual workload further increased, the burst of information could lead to tunnel vision, consequently reducing saccade amplitude, which made the subjects’ visual effort difficult to cope with. In previous studies, the saccade amplitude has been found to be significantly decreased during air traffic controller operations with demanding workloads and complex tone counting tasks [70,71]. Therefore, task performance initially improved when optimal visual search efficiency was achieved under the MMW, and subsequently declined as a result of the severe stress and effort demands resulting from the HMW.

Additionally, there are several confounding factors to consider when interpreting the results of this study. The duration of tasks could affect MW and task performance. A laboratory study [72] investigated the effect of task duration on productivity, safety, and user satisfaction during proofreading tasks. The study showed that with the increase in task duration, user’s perceived visual discomfort and visual fatigue, proofreading task speed, and MW increased significantly, while proofreading task accuracy and user satisfaction decreased significantly. The researchers suggested that short breaks were needed between each 15-min proofreading task to improve task performance and visual health. In our study, the subjects were asked to rest for 5 min after each 12-min MATB task to avoid the cumulative effect of fatigue. Several studies have indicated that personality and individual sensitivity may also influence the physiological response to noise. Park et al. [73] found that high noise sensitive individuals showed more noise annoyance than low noise sensitive individuals. Subjects with high noise sensitivity also showed more obvious physiological responses to noise. Golmohammadi et al. [74] reported that individual and personality variables could mediate or modulate the level of perceived and psychological effects induced by occupational noise exposure. Abbasi et al. [75] studied the effects of low- and high-frequency noise on sensitivity, annoyance and loudness perception in relation to personality traits. They suggested that personality traits could affect noise perception, especially for high-frequency noise exposure. Age is also a key factor that may exacerbate the adverse effects on cognitive performance. Boele-Vos et al. [76] found that older cyclists had lower reaction times and hit rates than younger cyclists. Older cyclists experience increased mental load during complex tasks, especially cycling uphill. A study of private pilots’ cognitive performance showed that older pilots had impaired performance relative to younger pilots, with the greatest impairments observed during the highest-load spatial working memory tasks [77]. Cantin et al. [78] also found that older drivers showed longer reaction times than younger drivers. That is, driving caused older drivers to have a greater MW than younger drivers, and the more complex driving environment exacerbated this effect. These studies supported the existence of age-related decline in cognitive function and complex task performance.

### 4.2. Physiological Mechanisms Affecting Task Performance

The mechanisms underlying psychophysiological responses to stress [79] might be used to explain how noise exposure and MW affect physiological indicators. When the body perceives stressors, the initial coping mode for dealing with stress is through the aggressive fight-or-flight response. When the body is under stress, the defenses and behavioral arousal of the physiological system are activated. The increased levels of stress favor the body’s defense responses, which are manifested as additional physiological responses, including elevated HRV, increased theta relative power, constricted pupil diameter, and decreased saccade amplitude. As stress levels continue to rise, the body shifts from active defense to passive self-protection (defeat response) to avoid being overwhelmed and exhausted. In this study, the subjects showed the best MATB task performance under the MMW. The aforementioned physiological mechanisms could explain this inverted U-shaped relationship between MW and task performance. As the MW increases, an enhanced physiological stress response is conducive to improving task performance, until optimum levels of arousal are achieved with moderate workloads. However, excessive stress and demanding effort from the HMW could then lead to decreased task performance [80]. This is demonstrated by the physiological responses to increased MW, as stated in Section 3.2.

**Figure 10:**
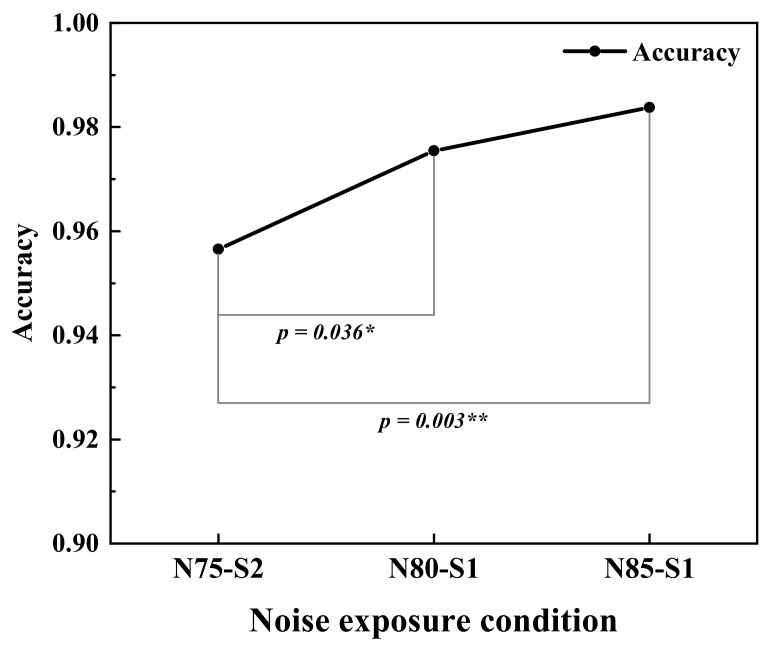
Accuracy of MATB tasks under three noise exposure conditions. * (*p* < 0.05), ** (*p* < 0.01).

No significant effect of elevated noise sharpness was found on response time and weighted task performance. However, the increased sharpness could result in significantly lower task accuracy, as shown in Figure 10. The alpha relative power was modestly lower under N75-S2 than under N80-S1. The mean pupil diameter and the saccade amplitude were also slightly increased with elevated sharpness. These results indicated a higher stress level and increased visual search effort in response to higher noise sharpness. The body must devote additional attentional resources in response to the detrimental effects of deteriorating sound quality, which reduces the attentional resources dedicated to performing tasks [81]. It was also concluded that when disturbed by noise, the body often tries to overcome these distractions by putting in more effort and redistributing attention [82,83]. Therefore, task performance could be negatively impacted when physiological regulation struggles to cope with distractions from increased sharpness.

### 4.3. Limitation

There are several limitations of this study, which are as follows: (1) Physiological measurements were limited to cerebral, cardiovascular, and visual parameters. More indicators, such as respiration, cortisol, blood pressure, and electromyogram, should be measured to thoroughly investigate the physiological responses. (2) This study only considered noise sharpness as an indicator of sound quality. More sound quality parameters, such as loudness, roughness, and psychoacoustic annoyance, ought to be assessed in future studies. (3) Only three noise exposure conditions were set up due to limited experimental resources. More experimental conditions with a wider SPL range and more sound quality parameters should be implemented in future studies. (4) The recruited subjects were limited to healthy male students, as the research target population was operators in high-load work conditions. Thus, the confounding effects of personal factors (age, gender, personality, and individual sensitivity, etc.) should be better considered in future studies.

## 5. Conclusions

The purpose of the study was to examine the physiological response to various noise exposure conditions and MW levels during MATB task execution. The main findings are summarized as follows:

(1) The physiological responses during the task tests were more significant than those at rest, indicating that additional effort was needed to cope with the adverse conditions when executing the tasks.
(2) The subjects showed increased HRV metrics, modestly lower alpha relative power of EEG, and more visual search effort spent when they accomplished the tasks at higher SPL or increased noise sharpness. It can be inferred that the subjects had to put in more effort to cope with the detrimental effects of unfavorable noise exposure.
(3) The elevated MW resulted in higher theta relative power of EEG and decreased average saccade velocity. This indicated that the subjects were more stressed and it took more visual search effort to perform tasks as the MW increased. Either high or low MW was related to reduced saccade amplitude and decreased task performance, which could help explain the inverted U-shaped relationship between MW and task performance.

In sum, the research results suggest that severe noise exposure and unsatisfactory MW can trigger physiological regulation that could further influence the stress response and task performance.

## Figures and Tables

**Table 1:** Specifications of the indoor environmental quality tester MI6401.

Thermal Environment Parameters	Measurement Range	Accuracy	Resolution
Air temperature	−20 °C–+60 °C	±0.2 °C	0.1 °C
Relative humidity	0% RH–10% RH	±3% RH	0.1% RH
	10% RH–90% RH	±2% RH	0.1% RH
	90% RH–100% RH	±3% RH	0.1% RH
Air velocity	0.1 m/s–9.9 m/s	±(0.05 m/s + 5%)	0.01 m/s
	10.0 m/s–20.0 m/s	±(5%)	0.1 m/s
Black bulb temperature	10.0 °C–49.9 °C	±0.5 °C	0.1 °C
	50.0 °C–84.9 °C	±1.0 °C	0.1 °C
	85.0 °C–120.0 °C	±1.5 °C	0.1 °C

**Table 2:** Measured environmental parameters under three noise exposure conditions.

Environment Parameters	N85-S1	N80-S1	N75-S2
A-weighted SPL (dB(A))	84.2 ± 0.8	78.3 ± 0.7	75.0 ± 0.8
Sharpness (acum)	1.28 ± 0.03	1.30 ± 0.03	2.42 ± 0.04
Air temperature (°C)	20.9 ± 0.6	21.0 ± 0.6	21.1 ± 0.6
Relative humidity (%)	26.5 ± 8.1	29.0 ± 7.5	24.7 ± 5.6
Air velocity (m/s)	0.12 ± 0.01	0.13 ± 0.01	0.12 ± 0.01
Black bulb temperature (°C)	21.1 ± 0.6	21.2 ± 0.6	21.3 ± 0.5

**Table 3:** Measured metrics of the physiological parameters.

Parameter	Metric	Implication
ECG	SDNN (ms)	Standard deviation of NN interval time series
	RMSSD (ms)	Root mean square of successive differences between normal heartbeats
	pNN50 (%)	Percentage of successive RR intervals that differ by more than 50 ms
EEG	Alpha relative power	Inability to focus, relaxation (8–13 Hz)
	Beta relative power	High arousal, stress (14–30 Hz)
	Theta relative power	Drowsy, depression (4–7 Hz)
	Delta relative power	Extreme fatigue, deep sleep (1–3 Hz)
Eye movements	Fixation duration (ms)	The sum of time the eyes remain fixated on each fixation point in the task scene region
	Saccade amplitude (px)	The distance from the end of the last fixation to the beginning of the next fixation in pixels
	Saccade velocity (px/ms)	The average distance per second between fixation points, defined as the saccade amplitude divided by the saccade time
	Mean pupil diameter (px)	The average diameter of left and right pupil

**Table 4:** GAMM results of noise exposure and MW effects on ECG metrics during task execution. * (*p* < 0.05), ** (*p* < 0.01).

ECG Metrics		Noise Exposure			MW			Intercept
		N75-S2	N80-S1	N85-S1	LMW	MMW	HMW	
SDNN (ms)	Estimate	−14.380 *	−9.834	Reference	Reference	1.806	1.847	78.858
	*p*	**0.016**	0.100			0.755	0.761	-
RMSSD (ms)	Estimate	−6.443	−9.721			7.517	7.815	52.715
	*p*	0.310	0.142			0.223	0.232	-
pNN50 (%)	Estimate	−0.124 **	−0.076 *			0.018	0.036	0.302
	*p*	**<0.001**	**0.029**			0.600	0.302	-
SDNN (ms)	Estimate	−4.541	Reference	9.834	−1.806	Reference	0.041	70.825
	*p*	0.451		0.100	0.755		0.995	-
RMSSD (ms)	Estimate	3.278		9.721	−7.517		0.298	50.512
	*p*	0.611		0.142	0.223		0.964	-
pNN50 (%)	Estimate	−0.048		0.076 *	−0.018		0.018	0.244
	*p*	0.172		**0.029**	0.600		0.595	-

**Table 5:** GAMM results of noise exposure and MW effects on EEG metrics during task execution. ** (*p* < 0.01).

EEG Metrics		Noise Exposure			MW			Intercept
		N75-S2	N80-S1	N85-S1	LMW	MMW	HMW	
Alpha relative power	Estimate	0.002	−0.006	Reference	Reference	0.002	−0.006	0.164
	*p*	0.589	0.190			0.680	0.188	-
Beta relative power	Estimate	0.004	0.005			0.000	−0.001	0.067
	*p*	0.322	0.193			0.963	0.713	-
Theta relative power	Estimate	0.001	0.000			0.001	0.011 **	0.191
	*p*	0.742	0.772			0.721	**0.002**	-
Delta relative power	Estimate	−0.004	0.004			0.000	0.002	0.555
	*p*	0.588	0.638			0.971	0.784	-
Alpha relative power	Estimate	0.008	Reference	0.006	−0.002	Reference	−0.008	0.160
	*p*	0.066		0.190	0.680		0.085	-
Beta relative power	Estimate	−0.001		−0.005	0.000		−0.002	0.072
	*p*	0.753		0.193	0.963		0.679	-
Theta relative power	Estimate	0.002		0.000	−0.001		0.009 **	0.192
	*p*	0.537		0.772	0.721		**0.007**	-
Delta relative power	Estimate	−0.008		−0.004	0.000		0.002	0.558
	*p*	0.312		0.638	0.971		0.756	-

**Table 6:** GAMM results of noise exposure and MW effects on eye movement metrics during task execution. * (*p* < 0.05), ** (*p* < 0.01).

Eye Movements		Noise Exposure			MW			Intercept
		N75-S2	N80-S1	N85-S1	LMW	MMW	HMW	
Fixation duration	Estimate	37.886	3.089	Reference	Reference	−28.991	−39.680	455.403
	*p*	0.165	0.909			0.287	0.146	
Saccade amplitude	Estimate	18.092	7.504			38.851 **	20.548 *	329.040
	*p*	0.053	0.418			**<0.001**	**0.028**	
Saccade velocity	Estimate	−0.015	−0.009			−0.025	−0.049 **	1.417
	*p*	0.364	0.572			0.127	**0.003**	
Mean pupil diameter	Estimate	−0.371	−0.675 *			0.034	0.285	18.549
	*p*	0.153	**0.010**			0.894	0.271	
Fixation duration	Estimate	34.796	Reference	−3.089	28.991	Reference	−10.688	455.403
	*p*	0.202		0.909	0.287		0.694	
Saccade amplitude	Estimate	10.588		−7.504	38.851 **		−18.303 *	375.395
	*p*	0.254		0.418	**<0.001**		**0.050**	
Saccade velocity	Estimate	−0.006		0.009	0.025		−0.024	1.383
	*p*	0.730		0.572	0.127		0.130	
Mean pupil diameter	Estimate	0.304		0.675*	−0.034		0.251	17.908
	*p*	0.240		**0.010**	0.894		0.333	

## Data Availability

The data presented in this study are available upon request from the corresponding author.
